# Can We Boost Treatment Adherence to an Online Transdiagnostic Intervention by Adding Self-Enhancement Strategies? Results From a Randomized Controlled Non-inferiority Trial

**DOI:** 10.3389/fpsyg.2021.752249

**Published:** 2021-12-02

**Authors:** Andreea Bogdana Isbăşoiu, Bogdan Tudor Tulbure, Andrei Rusu, Florin Alin Sava

**Affiliations:** Department of Psychology, West University of Timişoara, Timişoara, Romania

**Keywords:** transdiagnostic, anxiety, depression, unified protocol, self-enhance, treatment adherence, non-inferiority trial

## Abstract

**Background:** Internet-delivered psychotherapy represents an impactful large-scale solution for addressing psychological disorders. In spite of its flexibility and scalability, the fact that the ones in need have to initiate and sustain the curse of the treatment by themselves comes with considerable downsides in terms of treatment adherence. One solution could be to increase the ease of use and attractivity of the strategies and assignments from such programs. The present study aims to address this issue by incorporating a series of self-oriented strategies to the validated internet-delivered short version of the Unified Protocol (UP). By this mean we intend to complement the symptom-focused assignments, which may be more suitable in a therapist assisted context, with ones designed for self-enhancement, which may be easier approached as self-initiated. Based on a randomized controlled non-inferiority trial we compared the modified version of the UP with the standard short version.

**Method:** The trial design was factorial, with two parallel arms and three measurement moments (baseline, post-intervention and 6-months follow-up). A total of 284 participants were randomly assigned to the intervention or the active control groups. The intervention group (baseline *n* = 142) received the self-enhanced nine modules of the UP (Self-enhanced 9UP) while the active control (baseline *n* = 142) received the standard nine modules (9UP). The newly added techniques were inspired by the acceptance and commitment therapy and were specific for self-concepts such as self-compassion or unconditional self-acceptance. Both programs lasted for 9 weeks. The non-inferiority of the Self-enhanced 9UP was tested against a margin of *d* = −0.35, on the following primary outcome measures: Patient Health Questionnaire 9 (PHQ9) – operationalization for depression; Generalized Anxiety Disorder 7 (GAD7) – operationalization for generalized anxiety or worry; Social Phobia Inventory (SPIN) – operationalization for social phobia; and Panic Disorder Severity Scale-Self Report (PDSS-SR) – that showed participants’ level of panic. Treatment adherence was assessed through the drop-out analyses and the engagement in completing the homework assignments. Secondary outcome measures included several self-concept measures: Self-Compassion Scale (SCS); Rosenberg Self-Esteem Scale (RSES); Unconditional Self-Acceptance Questionnaire (USAQ); New General Self-Efficacy Scale (NGSE); and Self-Concept Clarity Scale (SCCS). On the secondary outcomes we explored the potential boost of effectiveness produced by the newly added self-enhancement components.

**Results:** The dropout rates were similar in both groups (approximately 45%) and high overall. Adherence to treatment assignments was also modest and similar between groups (on average participants completed approximately half of the tasks), without a statistically significant bias toward the self-enhancement ones. Overall, both the intention-to-treat and completers analyses yielded no significant group by time interactions for any of the post-intervention and follow-up measurements, but a few non-inferiority analyses suggested that the Self-enhanced 9UP had a significantly weaker effectiveness than the standard 9UP. Within-group analyses showed significant alleviations on all the primary and secondary outcomes for both groups. The effect size estimates were mainly medium and high, and their magnitude tended to be kept also at 6-months follow-up.

**Discussion:** We failed to increase treatment adherence, but we found support with some exceptions, for the non-inferiority hypothesis. Hence, the alterations performed to the 9UP protocol, although they did not boost the treatment attractiveness, they also did not decrease the treatment effectiveness as suggested by most non-inferiority analyses. Likewise, the gain on self-concepts was produced by both groups. Hence, the short version of the UP seems to have the potential of effectively alleviating a larger palette of psychological variables associated with mental health symptoms than previously known. Even though our main objective was only partially achieved, these secondary results are insightful and could open new avenues of research.

**Clinical Trial Registration:** This trial has been registered at ClinicalTrials.Gov (NCT03917550; 17 April 2019; https://clinicaltrials.gov/ct2/show/NCT03917550).

## Introduction

Anxiety and depression are some of the most common mental disorders among adults. Worldwide, a total of 322 million people live with depression, and an additional 264 million live with anxiety (World Health Organization, 2017). Unfortunately, compared to 10 years before (i.e., 2005) some of the most recent epidemiological estimates (Walker et al., 2015) display an 18.4% increase for depression and 14.9% for anxiety. Such increases are only partially explained by population growth and aging, contributing to significant impairments in health and functional status. The data speak by themselves and point toward the constant need for evidence-based and large-scale strategies that could effectively address anxiety and depression. In this context, the importance of internet interventions as unbounded solutions for offering psychological treatments has never been more momentous. Also, the restrictions imposed in fighting the world-wide COVID-19 pandemic revealed this type of approaches as some of the only means to reach for those in need of psychological assistance. Guided internet-delivered therapy has plenty of advantages regarding its accessibility and flexibility, and has already shown promising results (see Berger et al., 2009; Furmark et al., 2009; Titov et al., 2011; Johansson and Andersson, 2012; Andersson, 2014; Tulbure et al., 2018; to name just a few randomized controlled trials). However, participants’ high empowerment over the treatment process translates into self-regulatory effort (Donkin and Glozier, 2012; Zarski et al., 2018), thus impacting their adherence to such treatments (Beatty and Binnion, 2016; Flett et al., 2019; Arndt et al., 2020). Adherence reflects the degree to which participants receive the “active ingredients” of the program (Danaher et al., 2006), and was supported to be a significant mediator toward treatment outcomes (Arndt et al., 2020). Hence, the higher the adherence to the protocol, the higher the resulting mental health alleviation. Therefore, understanding and fostering treatment adherence are necessary and current concerns for the research on internet interventions (Zarski et al., 2018; Arndt et al., 2020).

Likewise, transdiagnostic interventions raised as a response to the issue of alarmingly high comorbidity rates between psychological disorders (Schaeuffele et al., 2021). This class of therapies is being designed to address transdiagnostic processes, i.e., psychological mechanisms which are common sources for the onset and/or development of several types of psychopathologies (Harvey et al., 2004). The Unified Protocol for Transdiagnostic Treatment of Emotional Disorders (UP; Barlow et al., 2011) is such a successful example, and is focused on emotion regulation as a shared mechanism (Sakiris and Berle, 2019). Moreover, recent findings also reported changes in neuroticism following UP (Zemestani et al., 2021), a personality dimension which is associated with the experience of intense negative emotions and a well-documented pathological risk factor (Kalokerinos et al., 2020). The protocol’s efficacy, effectiveness and generalizability already rely on a large and robust body of evidence (see Sakiris and Berle, 2019; Cassiello-Robbins et al., 2020 for reviews and meta-analyses). The most recent systematic review (Cassiello-Robbins et al., 2020) identified 77 studies indicating support for the UP’s suitability to a wide range of psychological conditions (i.e., 9 types of disorders), and also non-diagnosable problems.

Recently, Tulbure et al. (2018) delivered a shorter version of the UP that consisted of 9 web-based sessions (9UP) and found that relative to the wait-list control group, the intervention yielded medium to large effect sizes for the primary (e.g., anxiety, depression) and secondary (e.g., anxiety sensitivity) outcome measures. The effects were measured immediately after the treatment and 6 months later. Moreover, the intervention was perceived as credible, and participants declared themselves to be satisfied with the program. But in spite of these results, the modest treatment adherence reflected the status quo of internet interventions. For example, from the maximum of homework assignments, on average, participants completed a bit less than 50% of them. This observation occurred in spite of the fact that the program was not entirely self-help, but a therapist guided internet intervention. Importantly, each participant’s number of finished assignments correlated negatively with the number of clinical disorders diagnosed at post-treatment (*r* = –0.23). Therefore, together with previous findings and observations regarding adherence to internet interventions (e.g., Zarski et al., 2018; Arndt et al., 2020), this particular result had a pivotal role in the development of the current study.

The present study aims to increase the adherence to an already validated guided internet-delivered psychological treatment, namely the online version (Tulbure et al., 2018) of the Unified Protocol for Transdiagnostic Treatment of Emotional Disorders (UP; Barlow et al., 2011). Based on a two-armed randomized non-inferiority trial we compared a modified version of the internet-based UP with the protocol as validated by Tulbure et al. (2018). Since treatment adherence to internet interventions can be validly operationalized through the extent in which participants complete their assignments (Tulbure et al., 2018), the modifications that we came up with targeted this part of the program. We altered or replaced some non-critical assignments with newer ones derived from the self-enhancement literature. Through this modification, we tried to complement the mainly symptom-focused assignments of the UP, which may be better suited in a therapist delivered format, with more self-oriented ones, which may be easier approached in a standalone manner. Likewise, such self-enhancement add-on therapeutic techniques could bring the participants in deeper contact with their inner structure and lead to a greater desire to complete homework assignments and to progress further on the path of their own healing.

Since a rather neglected aspect in the transdiagnostic interventions for affective and anxiety disorders is how clients see themselves at a deeper level (their self-concept), the add-ons to the web-based UP protocol were psychotherapeutic techniques which aimed to bring participants in deeper contact with their inner structure. The rationale was that this could lead to a greater desire to achieve their homework and thus to progress further on the path of their own healing. Such self-enhancement strategies could not only improve treatment adherence in the general medical context (Sirois and Hirsch, 2019) and the mental health context (e.g., Uzer-Kremers et al., 2020) but could also be beneficial for the healing progress. Higher levels of self-compassion and self-esteem generally lead to lower levels of stress and burden and more perceived internal resources to cope with stressors. These, in turn, are likely to reduce the symptoms of anxiety and depression. There were 9 modifications to the 9 UP modules, one for each module. As to avoid any impact on the effectiveness of the original program, and not to alter its length and structure, the new assignments only replaced redundant or too extensive explanations from the existing ones or, where the existent exercises were simple and intuitive, replaced the examples from the explanations. Hence, in order to make room for the newer exercises, we altered the original content of the protocol by making the requirements of the existent exercises and assignments more concise.

The idea of “self as a context” was incorporated in Acceptance and Commitment Therapy (ACT; Hayes and Strosahl, 2004), thus the new exercises that we added were inspired and picked from this area (e.g., Lim et al., 2005; Leahy et al., 2011). Moreover, we considered that since self-restructuring represents an integral part of emotion-regulation, enhancing self-concepts within the framework of the UP has the potential to easily mold on the existing protocol, and through the nature of the tasks, to improve treatment adherence. In summary, the new techniques aimed the following constructs: Gratitude and ways to cultivate it – Grounds for gratitude (module 1); Altruism and generosity toward others – Planning acts of generosity (module 2); Self-compassion – Validation of the compassionate self (modules 3 and 4); Unconditional self-acceptance – Diffusion practices for everyday life (modules 5 and 6); Self-esteem – Identify ineffective rules and assumptions (modules 7 and 8); Life after treatment – Recognition of achievements and future plans (module 9). As compared to the mainly symptom focused assignments of the UP, we expected that the more directly the assignments will put participants in contact with their own selves, the more their involvement in the therapeutic process would be, and thus would increase their overall engagement with the protocol of the intervention.

Therefore, since we performed (subtle) alterations to each of the nine modules of the short web based UP protocol, our first aim was to ensure that these modifications will not deteriorate the effectiveness of the intervention. Hence, using a two-armed randomized non-inferiority trial design, the present study compared the modified version (self-enhanced) of the 9UP with the validated internet delivered protocol (Tulbure et al., 2018) as active control. Our main prediction was that both groups produce significant alleviations of the outcomes and that the self-enhanced 9UP is non-inferior to the 9UP on all primary outcomes (i.e., depression and anxiety symptoms). Moreover, due to the newly added self-related assignments, we also expected that participants engage in completing these new tasks to a higher frequency than the original ones. Finally, as a secondary, rather exploratory, objective, we tested if the self-enhanced 9UP produces significantly higher augmentations to the self-concepts targeted by the newly added exercises (e.g., self-esteem, self-compassion) than the standard protocol.

## Materials and Methods

### Trial Design

In order to test if the modified version of the web-based 9UP intervention (which we refer to as the *Self-enhanced 9UP*) is at least as effective as the validated one but with improvements in terms of participants’ adherence, the study was designed as a factorial randomized controlled non-inferiority trial (trial registration: ClinicalTrials.gov NCT03917550) with two parallel arms and three measurement moments (i.e., baseline, post-intervention, and 6-months follow-up). Participants were randomized with a 1:1 ratio to either (1) the Self-enhanced 9UP intervention group or (2) the standard 9UP active control. The trial was conducted as designed, without any unmanageable interferences caused by the online framework. In designing and reporting the trial we guided ourselves by the CONSORT standards (Boutron et al., 2017).

### Participants

Potential participants were recruited online through nationwide mass-media news and social-media posts (mainly Facebook). The trial was advertised as free of charge and voluntary. General information about the intervention and the university affiliation of the study team was presented on the study’s public website. The trial was open for the general public and participants were encouraged to maintain anonymity by creating and using a new (neutral) email account before registration. After reading and approving the informed consent (i.e., by a compulsory checkbox) interested participants registered for the study and received an anonymous ID (e.g., 1234abcd). Afterward, they were invited to complete a number of online self-report questionnaires for the self-assessment of their symptoms. During registration, the email addresses were checked for conformity while participants’ mobile phone numbers were collected as part of the screening. Except for the phone interview, conducted before the trial admission, and brief phone reminders during the program (i.e., totalizing about 5 min per participant), there were no other synchronous or face-to-face interactions with the participants.

The inclusion criteria were as follows: (a) to be fluent in Romanian; (b) to be at least 18 years old; (c) to have basic computer/internet literacy, (d) to have at least a clinical diagnosis of major depressive disorder, and/or generalized anxiety disorder, and/or social anxiety disorder, and/or panic anxiety disorder and/or agoraphobia, and/or specific phobia or any combination of these conditions on Structured Clinical Interview for DSM-5^®^ (SCID 5). The exclusion criteria were as follows: (a) presence of suicidal plans; (b) changes in the dosage of psychotropic medication during the last month (if present); (c) presence of bipolar disorder or psychosis (according to medication status); (d) presence of post-traumatic stress disorder or obsessive-compulsive disorder (according to SCID 5 interview); (e) presence of an alcohol or substance abuse and/or dependence disorder; (f) current participation in other psychological programs or treatments; (g) presence of obvious obstacle to participate (i.e., no current Internet access, long travel plans during the treatment period, etc.).

Potential participants who met the inclusion criteria upon completing the web-based measures were further invited to take a phone interview based on SCID 5. After the interview, participants who met all the inclusion criteria were informed about their admission, while those who did not meet the inclusion criteria received a message regarding their status (with the main reason for their exclusion) and additional resources that could be helpful for their situation.

### Interventions

The main aim of this study was to directly compare two intervention strategies that comprised the two study arms: the UP for transdiagnostic treatment of anxiety and affective disorders that was originally developed by Barlow et al. (2011), in its short web-based 9 modules version (9UP; Tulbure et al., 2018), and a Self-enhanced 9UP version where enhancement strategies that address self-concepts were added. Both interventions were designed as stand-alone web-based programs that could address clinical symptoms of anxiety and affective disorder.

The central idea behind Barlow’s UP is that affective and anxiety disorders are caused by similar underlying mechanisms and that we could jointly address the common vulnerabilities. The UP comprised of the following therapeutic strategies: (1) encouraging emotional awareness by noticing emotional experiences and accepting intense emotions; (2) adopting flexible thinking strategies; (3) recognizing and changing the emotion driven behaviors (EDB); (4) facilitating emotional exposure (to interoceptive and situational threats). This short version of Barlow’s UP (9 web-based modules or the 9UP) that retained the essential ingredients of Barlow’s protocol was previously proven effective (see Tulbure et al., 2018; for details regarding this web-based intervention).

The ***9UP self-enhanced*** intervention also retained the essential components and homework tasks originally included in the UP but replaced the redundant tasks or reduced some extensive explanations with new homework tasks designed to address self-concepts such as self-esteem, self-compassion, or unconditional self-acceptance. The intervention strategies for enhancing self-concepts were based on previous literature (i.e., Lim et al., 2005; Leahy et al., 2011). An overview of the newly added applications is presented in Table 1.

**TABLE 1:** Overview of the newly added self-enhancement applications.

**Module**	**Self-enhancement add-on**	**Application (assignment)**	**Source of the application**
1	Gratitude and ways to cultivate it	Grounds for gratitude – finding reasons for being grateful in everyday life.	Leahy et al., 2011
2	Altruism and generosity towards others	Planning acts of generosity towards the others.	Leahy et al., 2011
3	Self-compassion and how can we improve it (part 1)	Imagining your compassionate self – description of the experience in a pleasant and relaxing place.	Leahy et al., 2011
4	Self-compassion and how can we improve it (part 2)	A letter of self-compassion – for the validation of the compassionate self.	Leahy et al., 2011
5	Unconditional self-acceptance and how can we improve it (part 1)	Diffusion practices for everyday life – a journal of behaviors based on unconditional acceptance and goodwill.	Leahy et al., 2011
6	Unconditional self-acceptance and how can we improve it (part 2)	Breaking identification with thoughts – keeping a diary of the daily “monsters.”	Leahy et al., 2011
7	Self-esteem and how can we improve it (part 1)	Journal of unrealistic and exaggerated expectations.	Lim et al., 2005
8	Self-esteem and how can we improve it (part 2)	Identifying the ineffective rules and assumptions.	Lim et al., 2005
9	Life after treatment. Recognition of achievements and future plans	Putting events in perspective: what could I still do?	Leahy et al., 2011

For both treatment arms the order of the sessions was retained form the original UP and the intervention content remained unchanged during the whole program, as no revisions or updates were provided during the trial. All participants were encouraged to be actively involved in the treatment by reading the information and completing the homework tasks for each week (as the optimal pace for the program). The amount of text per page was generally designed to fit an average laptop screen, requiring minimal scrolling. Both intervention arms consisted of nine modules (or web-based sessions) that were made available on a weekly basis, thus the 9-week total duration of the trial. Participants could access the intervention content whenever they decided to, and all previous modules remained activated and could be consulted at any time.

At the end of each week participants who completed their homework tasks received personalized feedback regarding the content of their homework. The feedback was delivered through an asynchronous internal email system that was designed to keep all messages within the secure web space of the therapy platform. If no homework was completed, participants received the following succession of messages starting from mid-week until they managed to complete or partially complete the tasks: two email reminders, two SMS reminders, and a short phone call (i.e., 5 min). Twelve graduate psychology students undergoing CBT training supervised by an experienced clinician assisted the participants throughout the study. Each graduate student was responsible for monitoring the evolution of a comparable number of participants from both treatment arms. We estimate that a graduate student spent on average 45 min per participant weekly. The written feedbacks were provided under the supervision of the experienced clinician who suggested response models and intervention strategies. If these web-based programs would be offered as routine programs in a clinical setting outside the university, we estimate that clinicians with average expertise would need about 20–25 min per participant per week.

### Outcomes

All primary and secondary outcome measures were administered online at pre-treatment, post-treatment, and at 6-months follow-up. A number of studies have shown that online questionnaires produce similar results as the classical pen-and-paper format (Hollandare et al., 2010). Moreover, the anxiety and depression measures were successfully used in previous web-based interventions implemented by our team (e.g., Tulbure et al., 2015, 2018).

Treatment adherence (the intensity or dosage of treatment use) was monitored by the sum of homework assignments completed by each participant. We used this operationalization since it correlated with the treatment effectiveness in the previous trial of the online 9UP (Tulbure et al., 2018). Moreover, we complemented these results with the drop-out analysis and participants’ feedback obtained through the online questionnaire at post-treatment (i.e., treatment satisfaction).

#### Primary Outcome Measures

*Patient Health Questionnaire 9* (PHQ9; Kroenke et al., 2001) was designed to measure participants’ level of depression reflecting the diagnostic criteria. The scale is one-dimensional and the total score ranges from 0 to 27. High scores are associated with elevated levels of depression. Reliability on our sample was at least optimal at each of the three measurement moments (α = [0.85,0.91]).

*Generalized Anxiety Disorder 7* (GAD7; Spitzer et al., 2006) was designed to measure participants’ level of generalized anxiety or worry also reflecting the diagnostic criteria. The scale is unidimensional and the total score ranges from 0 to 21. High scores reflect higher levels of worry. Reliability on our sample was at least optimal at each measurement moment (α = [0.88,0.94]).

*Social Phobia Inventory* (SPIN; Connor et al., 2000) was designed to measure participants’ level of social phobia. The scale is unidimensional and the total score ranges from 0 to 68. High scores are associated with high levels of social phobia. Reliability on our sample was excellent at each measurement moment (α = [0.93,0.95]).

*Panic Disorder Severity Scale-Self Report* (PDSS-SR; Shear et al., 2001) was designed to measure participants’ level of panic. The scale is unidimensional and the total score rages from 0 to 28. High scores are associated with elevated levels of panic. Reliability on our sample was excellent at each measurement moment (α = [0.92,0.93]).

#### Secondary Outcome Measures

*Self-Concept Clarity Scale* (SCCS; Campbell et al., 1996) was designed to measure participants’ Self-Concept Clarity. The scale is unidimensional and the total score rages from 12 to 60. High scores are associated with high levels of Self-Clarity. Reliability on our sample was at least optimal at each measurement moment (α = [0.88,0.91]).

*New General Self-Efficacy Scale* (NGSE; Chen et al., 2001) was designed to measure participants’ General Self-Efficacy. The scale is unidimensional and the total score rages from 8 to 40. High scores are associated with high levels of Self-Efficacy. Reliability on our sample was excellent at each measurement moment (α = [0.92,0.94]).

*Unconditional Self-Acceptance Questionnaire* (USAQ; Chamberlain and Haaga, 2001) was designed to measure participants’ level of Unconditional Self-Acceptance. The scale is unidimensional and the total score rages from 20 to 140. High scores are associated with high levels of unconditional Self-Acceptance. Reliability on our sample was at least optimal at each measurement moment (α = [0.86,0.88]).

*Rosenberg Self-Esteem Scale* (RSES; Rosenberg, 1965) was designed to measure participants’ level of Self-Esteem. The scale is unidimensional and the total score rages from 10 to 40. High scores are associated with high levels of Self-Esteem. Reliability on our sample was excellent at each measurement moment (α = [0.90,0.93]).

*Self-Compassion Scale* (SCS; Neff, 2003) was designed to measure participants’ level of their Self-Compassion. The scale is multidimensional and the total score ranges from 26 to 130. For the mainly exploratory purpose for this study, we used only the total self-compassion score. Low values are associated with low levels of Self-Compassion, while high scores are associated with high levels of Self-Compassion. Reliability on our sample was at least optimal at each measurement moment (α = [0.89,0.95]).

Besides the aforementioned measures, the list of secondary outcomes also included the Work and Social Adjustment Scale (WSAS; Mundt et al., 2002), which measures symptom interference; Beck Depression Inventory-II (BDI-II; Beck et al., 1996), and Overall Depression Severity and Impairment Scale (ODSIS, Bentley et al., 2014), also as measures of depression; Overall Anxiety Severity and Impairment Scale (OASIS; Norman et al., 2006), and Penn State Worry Questionnaire (PSWQ; Meyer et al., 1990), as operationalizations of anxiety symptoms; and Anxiety Sensitivity Index 16 (ASI16; Reiss et al., 1986). These data are not in the main focus of the current manuscript and will serve secondary analyses (e.g., mechanisms of change) which will be reported in future manuscripts. However, for transparency, we tested the intervention effects also on these secondary outcomes and reported them as Supplementary Material.

#### Treatment Credibility

*Treatment credibility* inquired participants’ expectations regarding treatment and was measured at baseline with five items previously used on RCTs for internet interventions (e.g., Tulbure et al., 2018). The items were: *How much sense do you think it makes the method of delivering a psychological treatment over the internet?; How confident are you that the program will help you to better manage your emotions?; Would you recommend this program to a friend with the same emotional difficulties?; How effective will this program be in managing other uncomfortable emotions?; How confident are you that you will make improvements at the end of this program?* The response format was based on a 10-point Liker scale (e.g., *0 – not at all vs. 10 – to a great extant*). Internal consistency of the five items was α = 0.88. Credibility scores could have ranged between 0 and 50, higher scores represent greater credibility.

### Sample Size

Our main goal was to test the non-inferiority hypothesis, namely that altering the content of 9UP in order to fit the extra self-related assignments will not decrease its effectiveness. Hence, we conducted the sample size estimation for a non-inferiority trial with continuous outcomes. Based on the online calculator (Sealed Envelope Ltd., 2012) developed after the recommendations of Julious (2004), for a 5% significance level, 80% power and a non-inferiority margin of Cohen’s *d* = 0.35, the sample size required per group is of 101 participants (total *N* = 202). Considering a 20% attrition rate, the sample aimed to be recruited was of 222 participants.

### Randomization and Blinding

Before starting the interventions, all included participants were randomly assigned by one of the authors (BTT), using a 1:1 ratio *via* a true random number generator^1^. No randomization restrictions were used. Included participants were then assigned to their intervention group by volunteering graduate students. Participants were the only actors blinded regarding the specific nature of the comparison (the non-inferiority hypothesis) as they were informed about the two treatments, but they were unaware which they will be receiving.

### Statistical Methods

In order to test our predictions, we applied several sets of analyses. With the aid of linear mixed model effects (LMM) we first tested for any significant differences between the two groups at post-intervention or follow-up (group by time interactions). The interaction between factors was treated as fixed effect and the varying intercept of participants was set as random. Also based on LMM we tested the within-group modifications (for each separate models). In this case we tested the baseline to post-intervention, and baseline to follow-up modifications in outcomes. As in the previous trial on 9UP (Tulbure et al., 2018), we included age, gender, and treatment credibility (which was assessed at baseline) as covariates in all LMM models. Also, these analyses were performed on all primary and secondary outcome measures and were conducted on the Intent-to-treat (ITT) principle. For each between or within comparison we reported the Cohen’s *d* effect size estimate and its 95% confidence interval.

Since statistically non-significant differences between two groups are not implying equivalence (or non-inferiority, as in our case), there are dedicated tests developed for investigating such effects. Basically, a non-inferiority analysis tests if a new intervention is not worse than the standard one by more than a pre-specified extent, which is called the ‘non-inferiority margin’. On continuous outcomes this bound can be specified as the smallest acceptable effect size. The standards for controlled trials developed by regulators (e.g., Committee for Medicinal Products for Human Use, 2005) suggest selecting the non-inferiority margin based on existing meta-analytical evidence from effectiveness trials on the active comparator and considering the lower limit of the 95% CI of the effect. However, this value should also be scrutinized by expert opinion as to ensure its clinical significance. In our case, we only had the previously conducted effectiveness trial on the web-based 9UP and the general meta-analyses on the unified protocol. The lower limit of the 95% CI for the effect size estimates on depression and anxiety outcomes was in the range of (0.30,0.40). Hence, we selected *d* = 0.35 (the mid-point between a small and medium effect) as the non-inferiority margin. We conducted the actual non-inferiority analyses based on equivalence *t*-tests for independent samples and reported if the observed data is significantly lower than the non-inferiority margin.

The analyses were carried out in R version 4.0.5 (R Core Team, 2021), with package *lme4* (Bates et al., 2015) to perform linear mixed model analyses, package *emmeans* (Lenth, 2020) to extract Estimated Marginal Means, and package *TOSTER* (Lakens, 2018) for the non-inferiority analysis.

### Ethics

Study ethical approval was granted by the University Ethics Committee (5792/28.02.2019) before study initiation.

## Results

### Participant Flow and Treatment Adherence

**FIGURE 1:**
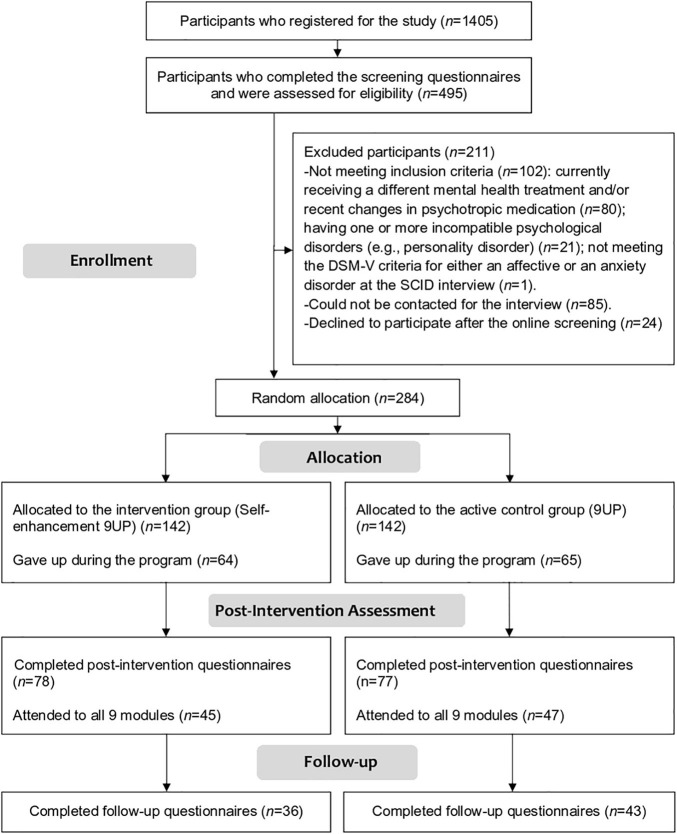
The flowchart displaying participants’ cycle throughout the study.

As illustrated in Figure 1, after mass-promoting the program, 1405 individuals manifested interest in participating. Out of them, 495 took part in the screening phase and 284 were found to be eligible and randomly allocated to the two arms of the study (*n* = 142 in each arm). During the 9 weeks of the program, both arms lost a similar number of people. Namely, from the intervention group 64 participants abandoned the program (45.07% dropout rate at post-intervention), and from the active control 65 participants (45.77% dropout rate at post-intervention). These numbers fail to meet our expectations (20% anticipated dropout), representing a more dramatic abandon rate than previously recorded in our internet interventions. Moreover, at six-moths follow-up responded only 36 of those who benefited of the Self-enhanced 9UP (74.65% dropout rate), and only 43 of participants from 9UP (69.72% drop-out rate).

Moreover, since it is required for the non-inferiority analysis, we also isolated the data per protocol (PP). Based on a more permissive approach, we considered all participants who took part to the entire intervention – all nine modules – by completing at least one homework assignment per module. There were 45 participants who completed at least one assignment per module of the Self-enhancement 9UP (31.69% protocol adherence), and 47 participants from the 9UP (33.09% protocol adherence).

Finally, as previously mentioned, our main operationalization for treatment adherence was the overall number of homework assignments completed by participants. Participants in the 9UP active control had 36 assignments while those from the Self-enhanced 9UP intervention had 45 (36 + 9 self-related tasks). On average, participants from the control condition completed 56.3% of their homework (*SD* = 35.0), and those who received the self-enhanced 9UP completed 61.8% of their homework (*SD* = 32.2). The difference between the two groups was not statistically significant, [*t*_(282)_ = 1.38, *p* = 0.084, one-tailed]. Hence, on average, participants of both groups managed to work only on approximately half the assignments (as also in the previous validation of the web-based 9UP; Tulbure et al., 2018). Most importantly, contrary to our prediction, participants from the Self-enhanced 9UP did not seem to be more attracted by the newly added tasks, manifesting a similar elaboration rate as for assignments of the active control group.

### Baseline Characteristics

Included participants (*N* = 284) had a mean age of 33.20 years old (*SD* = 10.09, age range 19–67) and most of them were women (*n* = 240, 84.5%). All participants had at least one clinical disorder (based on SCID I for DSM-5), and 32.04% of them received psychotherapy in the previous 4 years. On average, participants rated the treatment as rather credible (*M* = 39.05, *SD* = 7.65, range: 11–50). There were no statistically significant differences between the two groups on any of the recorded variables. Complete details regarding the screening characteristics of participants can be found in Table 2.

**TABLE 2:** Baseline characteristics of the participants in the two groups and the entire sample.

**Variable**	**Self-enhanced 9UP intervention (*n* = 142)**	**9UP active control (*n* = 142)**	**All participants (*n* = 284)**	**Statistic (df)**	***p***
Age (years)				*t* = 0.23 (282)	0.81
Mean (*SD*)	33.34(10.20)	33.06 (10.02)	33.20 (10.09)		
Gender, *n* (%)				χ^2^ = 0.43 (1)	0.51
Male	24 (16.9)	20 (14.1)	44 (15.5)		
Female	118 (83.1)	122 (85.9)	240 (84.5)		
Educational level, *n* (%)				χ^2^ = 0.94 (1)	0.53
Higher education	103 (72.53)	105 (73.94)	208 (73.24)		
High school or lower	39 (27.47)	37 (26.06)	76 (26.76)		
Marital status, *n* (%)				χ^2^ = 5.02 (4)	0.28
Never married	56 (39.43)	49 (34.5)	105 (36.97)		
In a relationship	29 (20.42)	24 (16.9)	53 (18.66)		
Married	40 (28.16)	54 (38.02)	94 (33.09)		
Divorced	15 (10.56)	15 (10.56)	30 (10.56)		
Widowed	2 (1.4)	0 (0.0)	2 (0.7)		
Primary diagnostic, *n* (%)				χ^2^ = 10.94 (9)	0.28
GAD	21 (14.78)	322(2.53)	53 (18.66)		
SAD	8 (5.63)	8 (5.63)	16 (5.63)		
MDD	41 (28.87)	38 (26.76)	79 (27.81)		
PD/A	26(18.3)	18(12.67)	44 (15.49)		
PDD	42(29.57)	43(30.28)	85 (29.92)		
Other	4 (2.81)	3 (2.11)	7 (2.46)		
Secondary diagnostic, *n* (%)				χ^2^ = 20.68(17)	0.24
GAD	46 (32.39)	38 (26.76)	84 (29.57)		
SAD	9 (6.33)	23 (16.19)	32 (11.26)		
MDD	7 (4.92)	14 (9.85)	21 (7.39)		
PD/A	28 (19.71)	24 (16.9)	52 (18.3)		
SP	1 (0.7)	1 (0.7)	2 (0.7)		
PDD	24 (16.9)	17 (11.97)	41 (14.43)		
Other	15 (10.56)	14 (9.85)	29 (10.21)		
Comorbid diagnostic, *n* (%)				χ^2^ = 55.71(19)	0.49
GAD	23 (16.19)	21 (14.78)	44 (15.49)		
SAD	12 (8.45)	12 (8.45)	24 (8.45)		
MDD	8 (5.63)	8 (5.63)	16 (5.63)		
PD/A	9 (6.33)	8 (5.63)	17 (5.98)		
PDD	9 (6.33)	7 (4.92)	16 (5.63)		
Other	8 (5.63)	12 (8.45)	20 (7.04)		
Previous psychotherapy (in the last 4 years), *n* (%)				χ^2^ = 0.14 (1)	0.70
Yes	47 (33.09)	44 (30.98)	91 (32.04)		
No	95 (66.9)	98 (69.01)	193 (67.95)		
Previous psychiatric diagnostic, *n* (%)				χ^2^ = 0.15 (1)	0.69
Yes	40 (28.16)	43 (30.28)	83 (29.22)		
No	102 (71.83)	99 (69.71)	201 (70.77)		
Currently under medication, *n* (%)				χ^2^ = 0.94 (1)	0.33
Yes	15 (10.56)	20 (14.08)	35 (12.32)		
No	127 (89.43)	122 (85.91)	249 (87.67)		
Time spent online (hours/day)				*t* = 1.17 (282)	0.24
Mean (*SD*)	4.67 (3.21)	4.26 (2.71)	4.46 (2.69)		
Treatment credibility					
Mean (*SD*)	38.91 (7.79)	39.19 (7.52)	39.05 (7.65)	*t* = 0.29 (279)	0.77

*GAD, generalized anxiety disorder; SAD, social anxiety disorder; MDD, major depressive disorder; PD/A, panic disorder/agoraphobia; PTSD, post-traumatic stress disorder; OCD, obsessive compulsive disorder; PDD, persistent depressive disorder.*

### Intervention Effects on Primary Outcomes

On the one hand, the LMM analyses revealed no significant group by time interactions on any of the primary outcomes, neither on baseline to post-intervention change nor on baseline to follow-up change (see Table 3 for detailed results). Hence, from this point of view, none of the two interventions were significantly more effective than the other (which should not be treated as non-inferiority). On the other hand, the decrease in symptoms within each group was statistically significant (all *p*s < 0.001; see Table 4 for detailed results). Both intervention programs (the 9UP and the Self-enhanced 9UP version) produced alleviations on all four primary outcomes, with effects size indices ranging from small to medium (for the Self-enhanced 9UP) and medium to large (for the 9UP active control) with overlapping confidence intervals between the two groups. Importantly, the effects kept a similar magnitude at follow-up.

**TABLE 3:** Estimated differences in mean change between baseline and post intervention, respectively, follow-up, for the intervention versus the control group (group by time interactions).

**Variable**	**Group × Time**	***b* [95% CI]**	t^*^ (df) ^†^	***d* [95% CI]**
*Primary outcomes*				
PHQ9	Post-intervention	0.47 [–1.37, 2.31]	0.50 (418)	–0.04 [–0.18, 0.11]
	Follow-up	1.45 [–1.27, 4.17]	1.05 (338)	–0.10 [–0.29, 0.09]
GAD7	Post-intervention	1.26 [–0.47, 2.99]	1.43 (415)	–0.10 [–0.24, 0.04]
	Follow-up	0.51 [–1.80, 2.83]	0.43 (336)	–0.04 [–0.21, 0.14]
SPIN	Post-intervention	2.96 [–0.91, 6.83]	1.50 (421)	–0.11 [–0.26, 0.03]
	Follow-up	3.80 [–1.67, 9.28]	1.36 (343)	–0.13 [–0.32, 0.06]
PDSS-SR	Post-intervention	1.30 [–0.20, 2.80]	1.70 (417)	–0.13 [–0.28, 0.02]
	Follow-up	0.76 [1.69, 3.22]	0.61 (338)	–0.06 [–0.27, 0.14]
*Secondary outcomes*				
SCCS	Post-intervention	–1.13 [–4.30, 2.04]	−2.70(414)	–0.05 [–0.09, –0.19]
	Follow-up	–1.29 [–5.36, 2.78]	−0.62(336)	0.06 [–0.13, 0.26]
NGSE	Post-intervention	–1.59 [–3.88, 0.69]	−1.37(414)	0.10 [–0.05, 0.25]
	Follow-up	–0.09 [–3.42, 3.24]	−0.05(334)	0.01 [–0.20, 0.21]
USAQ	Post-intervention	0.75 [4.18, 5.68]	0.30 (414)	–0.02 [0.17, –0.12]
	Follow-up	–3.09 [–10.34, 4.16]	−0.83(333)	0.08 [–0.11, 0.28]
RSES	Post-intervention	–1.10 [–2.66, 0.46]	−1.38(418)	0.10 [–0.04, –0.25]
	Follow-up	–1.41 [–3.62, 0.79]	−1.26(336)	0.13 [–0.08, 0.34]
SCS	Post-intervention	–1.73 [–8.05, 4.58]	−0.54(414)	0.04 [0.10, –0.17]
	Follow-up	–1.36 [–9.63, 6.90]	−0.32(333)	0.03 [–0.14, 0.19]

*All *p*s > 0.05

*^†^Degrees of freedom vary between measures because participants were not forced to fill-in the entire set of scales (hence, some were occasionally skipped). All models were adjusted for three covariates (age, gender, and treatment credibility).*

*b, Mean change difference in treatment versus control group estimate; d, Cohen’s *d* for between-groups effects; PHQ9, Patient Health Questionnaire 9; GAD7, Generalized Anxiety Disorder 7; SPIN, Social Phobia Inventory; PDSS-SR, Panic Disorder Severity Scale-Self Report; SCCS, Self-Concept Clarity Scale; NGSE, New General Self-Efficacy Scale; USAQ, Unconditional Self-Acceptance Questionnaire; RSES, Rosenberg Self-Esteem Scale; SCS, Self-Compassion Scale.*

**TABLE 4:** Estimates of mean differences between baseline and post intervention, respectively, follow-up (within-group effects).

**Variable**	**Baseline vs. Time**	**Self-enhanced 9UP intervention**			**9UP active control**		
		***b* [95% CI]**	t^*^ (df) ^†^	***d* [95% CI]**	***b* [95% CI]**	t^*^ (df) ^†^	***d* [95% CI]**
*Primary outcomes*							
PHQ9	Post-intervention	–6.31 [–7.52, –5.10]	−10.23(243)	0.74 [0.55, 0.93]	–5.89 [–7.23, –4.56]	−8.66(238)	0.93 [0.71, 1.14]
	Follow-up	–7.75 [–9.36, –6.13]	−9.41(243)	0.56 [0.38, 0.74]	–6.34 [–8.21, –4.48]	−6.68(238)	0.84 [0.64, 1.04]
GAD7	Post-intervention	–6.09 [–7.27, –4.90]	−10.08(240)	0.72 [0.91, 0.53]	–4.78 [–5.91, –3.64]	−8.26(237)	0.86 [0.67, 1.06]
	Follow-up	–6.23 [–7.81, –4.65]	−7.74(240)	0.56 [0.37, 0.74]	–5.25 [–6.83, –3.66]	−6.50(237)	0.65 [0.47, 0.83]
SPIN	Post-intervention	–11.31 [–13.68, –8.94]	−9.34(245)	0.49 [0.31, 0.68]	–8.17 [–11.02, –5.32]	−5.62(243)	0.83 [0.63, 1.03]
	Follow-up	–14.88 [–17.99, –11.78]	−9.40(245)	0.46 [0.28, 0.64]	–10.68 [–14.61, –6.75]	−5.33(243)	0.83 [0.63, 1.03]
PDSS-SR	Post-intervention	–3.53 [–4.60, –2.46]	−6.47(243)	0.40 [0.21, 0.58]	–2.24 [–3.25, –1.22]	−4.33(238)	0.60 [0.40, 0.79]
	Follow-up	–4.31 [–5.71, –2.90]	−5.99(243)	0.45 [0.26, 0.64]	–3.57 [–4.99, –2.15]	−4.92(238)	0.55 [0.36, 0.74]
*Secondary outcomes*							
SCCS	Post-intervention	8.01 [6.08, 9.93]	8.16 (239)	0.50 [0.31, 0.68]	6.83 [4.48, 9.19]	5.68 (237)	0.72 [0.52, 0.91]
	Follow-up	10.45 [7.89, 13.01]	8.00 (239)	0.47 [0.29, 0.65]	9.23 [5.93, 12.52]	5.49 (237)	0.70 [0.51, 0.89]
NGSE	Post-intervention	6.51 [4.82, 8.20]	7.56 (238)	0.60 [0.40, 0.79]	4.87 [3.42, 6.33]	6.56 (236)	0.68 [0.48, 0.88]
	Follow-up	5.22 [2.96, 7.48]	4.53 (238)	0.44 [0.25, 0.62]	5.03 [2.97, 7.10]	4.78 (236)	0.39 [0.22, 0.57]
USAQ	Post-intervention	13.84 [10.42, 17.26]	7.93 (238)	0.74 [0.54, 0.94]	14.60 [11.16, 18.04]	8.32 (232)	0.71 [0.52, 0.91]
	Follow-up	17.30 [12.67, 21.93]	7.32 (238)	0.50 [0.31, 0.68]	14.57 [9.50, 19.64]	5.63 (232)	0.65 [0.46, 0.85]
RSES	Post-intervention	5.08 [4.01, 6.15]	9.32 (238)	0.67 [0.47, 0.87]	4.02 [2.95, 5.08]	7.39 (236)	0.84 [0.63, 1.05]
	Follow-up	5.87 [4.42, 7.32]	7.94 (238)	0.52 [0.33, 0.71]	4.68 [3.10, 6.25]	5.82 (236)	0.71 [0.52, 0.91]
SCS	Post-intervention	19.67 [15.52, 23.82]	9.30 (238)	0.70 [0.51, 0.88]	18.14 [13.83, 22.45]	8.25 (235)	0.80 [0.60, 0.99]
	Follow-up	20.65 [15.08, 26.22]	7.27 (238)	0.52 [0.35, 0.69]	20.03 [13.85, 26.21]	6.36 (235)	0.61 [0.43, 0.79]

*All *p*s > 0.05.

*^†^Degrees of freedom vary between measures because participants were not forced to fill-in the entire set of scale (hence, some were occasionally skipped). All models were adjusted for three covariates (age, gender, and treatment credibility).*

*d, Cohen’s *d* for within-group effects; PHQ9, Patient Health Questionnaire 9; GAD7, Generalized Anxiety Disorder 7; SPIN, Social Phobia Inventory; PDSS-SR, Panic Disorder Severity Scale-Self Report; SCCS, Self-Concept Clarity Scale; NGSE, New General Self-Efficacy Scale; USAQ, Unconditional Self-Acceptance Questionnaire; RSES, Rosenberg Self-Esteem Scale; SCS, Self-Compassion Scale.*

### Non-inferiority Analysis

We conducted the non-inferiority analyses on the primary outcomes based on three scenarios: (1) data per protocol (PP; only on those participants who took part to the entire intervention – all modules), (2) intent-to-treat (ITT; all randomized participants, with the last observation carried forwards), and also on (3) completers (participants who completed any number of sessions or modules and also the post-intervention assessment). Even though the comparative reporting of PP and ITT results represent the recommended practice (e.g., Committee for Medicinal Products for Human Use, 2005), because of the large difference between the PP population (max *n* = 92) and the ITT one (max *n* = 284) we decided to also include a group with intermediate level of treatment adherence (completers max *n* = 155).

As can be seen from Table 5, the non-inferiority t-tests on PP data suggest that the self-enhanced 9UP program was non-inferior on most analyses based on the four main outcomes (*p*s < 0.025). The ITT data support the non-inferiority hypothesis in all cases, whereas the PP and completers data support the non-inferiority hypothesis on the majority of outcomes and occasions. Hence, with a cautionary note in mind because of the few results which departs from a non-inferiority scenario, we incline toward concluding that the non-inferiority of the self-enhanced 9UP program is supported by the data.

**TABLE 5:** Between-groups *t*-test comparisons for non-inferiority on all primary outcomes against the non-inferiority margin of *d* = –0.35.

**Variable**	***Baseline vs. Time***	***t (df)***	P^*^	**95% CI**
*PP*				
PHQ9	Post-intervention	1.86 (89)	0.033	[–0.37,0.45]
	Follow-up	1.96 (46)	0.028	[–0.35,0.78]
GAD7	Post-intervention	3.26 (89)	<0.001	[–0.08,0.75]
	Follow-up	1.79 (46)	0.040	[–0.40,0.73]
SPIN	Post-intervention	3.43 (90)	<0.001	[–0.05,0.77]
	Follow-up	2.46 (48)	0.009	[–0.22,0.90]
PDSS-SR	Post-intervention	2.85 (88)	0.003	[–0.16,0.66]
	Follow-up	1.28 (47)	0.103	[–0.55,0.58]
*ITT*				
PHQ9	Post-intervention	2.79 (275)	0.003	[–0.25,0.22]
	Follow-up	3.31 (275)	<0.001	[–0.19,0.28]
GAD7	Post-intervention	3.74 (274)	<0.001	[–0.11,0.37]
	Follow-up	3.98 (274)	<0.001	[–0.11,0.37]
SPIN	Post-intervention	4.05 (276)	<0.001	[–0.10,0.37]
	Follow-up	4.43 (276)	<0.001	[–0.05,0.42]
PDSS-SR	Post-intervention	4.13 (274)	<0.001	[–0.09,0.38]
	Follow-up	3.98 (274)	<0.001	[–0.11,0.37]
*Completers*				
PHQ9	Post-intervention	1.87 (146)	0.032	[–0.37,0.28]
	Follow-up	2.88 (67)	<0.001	[–0.13,0.82]
GAD7	Post-intervention	3.16 (144)	<0.001	[–0.15,0.49]
	Follow-up	1.97 (66)	0.027	[–0.35,0.60]
SPIN	Post-intervention	3.42 (148)	<0.001	[–0.11,0.53]
	Follow-up	2.95 (71)	0.002	[–0.12,0.80]
PDSS-SR	Post-intervention	3.39 (145)	<0.001	[–0.12,0.53]
	Follow-up	1.77 (68)	0.041	[–0.39,0.55]

*Reference *p-*value for rejecting the null hypothesis = 0.025. PP, per protocol; ITT, intent-to-treat; Completers (participants who provided post-intervention data); PHQ9, Patient Health Questionnaire 9; GAD7, Generalized Anxiety Disorder 7; SPIN, Social Phobia Inventory; PDSS-SR, Panic Disorder Severity Scale-Self Report.

### Intervention Effects on Secondary Outcomes

The LMM analyses found no significant group by time interaction (all *p*s > 0.05) for any of the secondary outcomes. Again, the non-significant differences in changes between the two intervention groups were both for baseline to post-intervention and baseline to follow-up data (Table 3). When it comes to within-group comparisons, both the baseline to post-intervention and baseline to follow-up alleviations were statistically significant (all *p*s < 0.001) for both groups (Table 4).

Even though our second direction of analyses – to test whether the use of self-oriented exercises lead to increases in the self-concept – was rather exploratory, the data seem not to support it. Not only that the differences between the two groups were not significant, but both produced positive modifications. In addition, the effect sizes on secondary outcomes for the self-enhanced 9UP program were on average smaller with *d* = 0.11 than the effect sizes for the 9UP program.

### Treatment Satisfaction

Treatment satisfaction was operationalized through nine questions addressed at post-intervention (see the complete list in Table 6). There were no significant differences between participants’ answers on any of the items. Overall, participants declared to be satisfied with the program. On the majority of the items the mean responses were close to the positive end of the scale. Notably, participants reported to have understood approximately 7 out of 9 modules (in both programs), spent almost 5 h per week on the program, and the activity was somehow demanding. These suggest that the level of difficulty of both programs is elevated and may be one of the reasons behind the dramatic drop-out/reduced treatment adherence.

**TABLE 6:** Descriptive statistics for the treatment satisfaction items.

**No.**	**Outcome**	**Self-enhanced 9UP intervention *M(SD)***	**9UP active control *M(SD)***	***t (df)***	***p***
1	Overall, how satisfied are you with the treatment you received? (*1-very unsatisfied vs. 5-very satisfied*)	4.35 (0.75)	4.40 (0.70)	−0.42(139)	0.67
2	How would you evaluate the quality of the information that was provided? (*1-very weak vs. 5-very good*)	4.60 (0.92)	4.52 (0.97)	0.53 (139)	0.59
3	How satisfied were you with the timing of the treatment program? (*1-too short, 3-appropriate, 5-too long*)	2.54 (0.83)	2.58 (0.84)	−0.29(139)	0.76
4	How many modules did you fully understood? (*out of 9*)	7.01 (1.88)	7.16 (1.88)	−0.47(139)	0.63
5	Please, estimate the average number of hours you spent in a week on the program.	4.79 (4.29)	4.95 (4.98)	−0.20(133)	0.83
6	How demanding were the activities? (*1-very little vs. 4-very much*)	2.78 (0.65)	2.77 (0.75)	0.06 (139)	0.94
7	The program helped me approach my problems more effectively. (*1-not at all vs. 4-to a great extant*)	3.47 (0.55)	3.38 (0.63)	0.85 (139)	0.39
8	How logical seemed to you the method that we used? (*0-not at all vs. 10-very logical*)	8.25 (1.89)	8.08 (2.07)	0.50 (139)	0.61
9	How confident would you be to recommend this method of treatment? (*0-not at all vs. 10-very confident*)	8.52 (1.77)	7.94 (2.67)	1.52 (113)	0.13

## Discussion

Conceptually the UP offers a solid third-wave cognitive-behavioral framework that seemed inviting for other researchers to test additional hypothesis (Sauer-Zavala et al., 2019). Our main goal in this study was to explore some alternative modalities to foster treatment adherence by introducing a new set of tasks derived from the self-enhancement literature. We compared the 9UP (active control arm) with a self-enhanced version of it (intervention arm) that included explicit intervention strategies designed for self-enhancement. More specifically, we hypothesized that the self-enhanced 9UP version will provide ampler space for self-talk, and therefore participants will adhere more to the treatment and maybe will be more satisfied by it compared to the 9UP. At the same time, we wanted to be assured that the modifications that we made to the 9UP protocol in order to fit the newer elements (some content elimination from the 9UP program to compensate for one new assignment for each module in the self-enhancement 9UP) will not significantly worsen the program.

Overall, both groups produced significant increases on each primary and secondary outcomes, with effect size estimate generally ranging from medium to high, and these positive effects preserved also at 6-months follow-up. However, when it comes to the particular objectives which we aimed through this study, the data were not in their favor. We were unable to find differences between the two groups in terms of treatment adherence. Specifically, the dropout rate recorded in our study was higher than previously recorded on 9UP (Tulbure et al., 2018) and similar for both groups (i.e., almost half the sample was lost at post-intervention and another 20% at follow-up). Also, adherence to assignments din not increase, participants completed on average a bit more than 50% of the tasks, without a predilection to complete the self-related ones. A possible explanation of the high drop-out rate comes from the treatment satisfaction items. Even though participants of both groups reported to be satisfied with the programs, they also suggested that these are rather difficult and demanding. As already pointed out in the literature on internet-interventions, the high self-regulatory effort needed from behalf of the participants is one key hindering factor when it comes to their adherence to such treatments (Donkin and Glozier, 2012; Beatty and Binnion, 2016; Zarski et al., 2018; Flett et al., 2019; Arndt et al., 2020). On this line of thought, it may be that adding the extra 9 add-ons to the UP, even though presumably more attractive and easily to follow, only increased the program’s complexity and participants’ workload. Another speculative explanation for the reduced treatment adherence is the dual (paradoxical) effect of self-enhancement. On the one hand, such exercises could increase participants’ comfort with the task and themselves, increasing the likeability of the training. On the other hand, self-boosting could increase individuals’ beliefs that they can handle day-to-day tasks without needing to rely on all prescribed exercises. Recent evidence (Harrell et al., 2021) supports this view, suggesting that people with low self-efficacy feel less equipped to face the burden they encounter. Therefore, they are more willing to engage in the training they could benefit from compared to people with high levels of self-efficacy. However, this result is discrepant from other findings (e.g., Sainsbury et al., 2018) that associated self-efficacy to better adherence in a different health context, dieting. Future studies should focus more on disentangling the role of various self-concepts such as self-esteem, self-efficacy, or self-compassion in treatment adherence. Likewise, future studies could also focus on the differential role of general and specific self-concepts. For instance, it might be the case that high levels of general self-efficacy and self-compassion could play a minor role in increasing treatment adherence, as the current research suggests. However, the situation would have been different if the focus were on specific self-concepts such as technology self-efficacy, self-regulatory self-efficacy, or specific self-compassion. These constructs seem highly relevant for internet-delivered treatments, demanding homework, and instances of adherence failure, respectively.

Moreover, since to some extent we altered the original UP protocol, we wanted to test whether these modifications will maintain its clinical impact (the non-inferiority hypothesis). The linear mixed model analyses revealed no significant differences between the two treatment arms. The specific non-inferiority analyses support these findings in all cases when conducting ITT analyses and in most cases when conducting data per protocol and completers analyses. We should take these results with caution, considering that the margin (*d* = 0.35) was not set on strong empirical grounds. If we accept the evidence supporting the non-inferiority hypothesis found in most cases, it appears that we were successful in making the 9UP more concise in order to add the new techniques in the self-enhancement arm, because its effectiveness was indeed not altered. The longer and sometimes redundant explanations in the standard 9UP program could have been eliminated to leave room for some self-enhancement exercises. Unfortunately, the non-inferiority gain was not associated with an increase in treatment adherence, making less useful our entire approach of altering the 9UP.

Finally, our second objective, which was rather exploratory, revealed not only that the self-enhancement strategies added to the 9UP did not improve or augmented the self-related constructs beyond the active control intervention, but also that the 9UP alone was quite successful in this regard. The data revealed that both interventions were able to improve participant’s self-concepts effectively, with medium to large ES, but the 9UP displayed slightly higher overall effects (with a couple of exceptions). These results are somehow surprising, considering that the 9UP does not explicitly address such self-concepts during the intervention and despite this fact, participants following the program were able to significantly improve them. It may be that an effective psychotherapy program like the UP and its shorter version (9UP) is able to positively impact participant’s self-concepts simply by developing cognitive flexibility skills and promoting avoidance reduction through exposure techniques. Due to its flexibility and focus on the functional nature of emotions, the UP could be used beyond the original purpose (symptoms reduction), being also beneficial for other purposes (e.g., personal development, self-enhancement). This aspect is important for the work of clinical psychologists. It seems it would be enough to correctly deliver the therapy protocol for the patients’ clinical condition. The self-constructs will follow the ascending path of the patient’s progress. Our role as psychotherapists remains to accompany the patient on this path of inner healing. Future research should investigate the mediators (processes) through which a 9UP transdiagnostic intervention also impacts on self-concepts and self-structures. It might be the case that cognitive restructuring and cognitive diffusion reduce self-criticism tendencies (Levin et al., 2018), which, in turn, facilitate self-enhancement.

Although taken separately the self-concepts demonstrated some improvements in specific intervention studies (Kirby et al., 2017; Wilson et al., 2018; Ferrari et al., 2019), when we addressed them simultaneously with the 9UP by means of brief, non-repetitive homework tasks, their potential prove to be less than we have hoped for. Probably the more consistent and better focused approach of the 9UP program was able to keep participants on track and lead them to deeper and more coherent changes, while the dual approach of the Self-enhanced 9UP program, although overall effective, was unable to go beyond its active control golden standard. Thus, our “self-enhanced” program proved to be less worthy of proudly wearing such a name. Finally, we should consider that, on the state-trait continuum, self-concepts lay closer to the less malleable trait extreme compared to the emotional symptoms, that characterize affective and anxiety disorders, that lay closer the more malleable state extreme.

### Study Limitations

The results presented above should be considered in the context of the study limitations.

First of all, by design the intervention content of the two study arms (the 9UP and the self-enhanced 9UP) were significantly overlapping (about 80%). This makes more difficult to highlight eventual differences between the two programs. We had little room for decreasing the level of overlapping between the two programs because of ethical reasons. Evidence-based interventions (i.e. a previously tested 9UP program) are required as active controls when dealing with a clinical sample.

Working with a clinical sample might also explain why we failed in enhancing treatment adherence. When people are confronted with a high level of distress, as in our clinical sample, participants focus heavily on symptoms reduction. In other contexts, such as personal development or working with subclinical samples, the focus would be less on symptoms’ levels, but on participants’ level of well-being and perceived self-enhancement. Therefore, maybe when working with a different sample (i.e., subclinical), participants’ focus would lean toward self-enhancement than on reducing the emotional burden, given that such participants do not face intense emotional burden. For clinical samples, instead, our results suggest that there is no reason to alter the 9UP program, and no need to add self-enhancement modules.

Another relevant limitation of the current study refers to the high degree of observed dropout, which was double than expected and affected statistical power for our hypotheses. Overall, participants who did not dropout spent almost 5 h per week on the program, and perceived the activity as rather demanding, but useful. However, such a high workload might also have affected the willingness of participants for doing more homework assignments, therefore influencing the level of treatment adherence in both groups. Future studies could tackle this issue of the workload amount for internet-delivered studies. Maybe another protocol that will prolong the duration of the same program with 50% (e.g., a 14-session UP instead of 9-UP), would decrease the homework assignment workload per week will lead to different results with regards to the potential benefits of adding self-enhancement content.

## Conclusion

Both 9UP programs are reliable and very useful internet-delivered transdiagnostic treatments. There were no statistically significant differences between the two intervention groups for any of the 17 outcome measurements, neither at post-test, nor at follow-up. All statistically significant differences existed only within each treatment group, showing the progress made by all participants throughout the therapeutic program. The beneficial effects on their mental state, the decrease of the symptoms from anxiety and depression and the raising of the self-parameters were preserved even at 6 months after the end of the interventions, for both groups. These conclusions are weakened by the study limitations mentioned above. However, despite multiple signs of non-inferiority, there are no reasons to alter the existing 9UP program because the introduction of the self-enhancement modules did not lead to the expected increase in treatment adherence. Moreover, improvements in secondary outcomes referring to self-structures were also found in the arm that did not include self-enhancement tools. This happens because after any correctly implemented psychological intervention, the patients will adapt to new life situations helped by the process they went through. These beneficial changes reflect the process of inner healing and adaptive functioning to the outside world.

## Data Availability Statement

The raw data supporting the conclusions of this article will be made available by the authors, without undue reservation.

## Ethics Statement

The studies involving human participants were reviewed and approved by the University Ethics Committee (5792/28.02.2019). The participants provided their written informed consent to participate in this study.

## Author Contributions

AI, FS, and BT contributed to the study design. AI developed the intervention program and supervised the study implementation. AR, BT, AI, and FS wrote the manuscript. All authors contributed to the article and approved the submitted version.

## Conflict of Interest

The authors declare that the research was conducted in the absence of any commercial or financial relationships that could be construed as a potential conflict of interest.

## Publisher’s Note

All claims expressed in this article are solely those of the authors and do not necessarily represent those of their affiliated organizations, or those of the publisher, the editors and the reviewers. Any product that may be evaluated in this article, or claim that may be made by its manufacturer, is not guaranteed or endorsed by the publisher.

## Footnotes

1
http://www.random.org
