# Supplementary material for: Can We Boost Treatment Adherence to an Online Transdiagnostic Intervention by Adding Self-Enhancement Strategies? Results From a Randomized Controlled Non-inferiority Trial
Source: Front Psychol. 2021 Dec 2;12:752249. doi: 10.3389/fpsyg.2021.752249 (PMC8675898; doi:10.3389/fpsyg.2021.752249)
Supplement: Supplementary file 1 [file Table_1.DOCX]

Supplementary Material

SM Table 1.

Estimated differences in mean change between baseline and post intervention*,* respectively, follow-up, for the intervention versus the control group (group by time interactions) for the secondary outcomes which fall outside of the aim of the present report.

| **Variable** | | **Group X Time** | | ***b* [95% CI]** | | ***t** *(df)*†** | | ***d* [95% CI]** | |
| --- | --- | --- | --- | --- | --- | --- | --- | --- | --- |
| *Secondary outcomes* | | | | | | | |  |  |
| WSAS | | Post-intervention | 0.56 [-2.22, 3.33] | 0.39 (416) | | -0.03 [-0.17, 0.11] | |  |  |
|  | | Follow-up | 0.56 [-3.42, 4.54] | 0.28 (336) | | -0.03 [-0.20, 0.15] | |  |  |
| BDI-II | | Post-intervention | 1.06 [-2.08, 4.21] | 0.66 (425) | | -0.05 [-0.19, 0.10] | |  |  |
|  | | Follow-up | 1.50 [-2.92, 5.93] | 0.67 (349) | | -0.06 [-0.25, 0.12] | |  |  |
| ODSIS | | Post-intervention | 0.16 [-1.10, 1.43] | 0.25 (418) | | -0.02 [-0.17, 0.13] | |  |  |
|  | | Follow-up | 0.14 [-1.97, 2.26] | 0.13 (337) | | -0.01 [-0.20, 0.17] | |  |  |
| OASIS | | Post-intervention | 0.18 [-0.96, 1.32] | 0.31 (418) | | -0.02 [-0.17, 0.13] | |  |  |
|  | | Follow-up | -0.73 [-2.51, 1.06] | -0.80 (338) | | 0.08 [-0.11, 0.26] | |  |  |
| PSWQ | | Post-intervention | 2.44 [-0.48, 5.37] | 1.64 (419) | | -0.12 [-0.26, -0.02] | |  |  |
|  | | Follow-up | 3.07 [-0.87, 7.02] | 1.53 (344) | | -0.14 [-0.31, 0.04] | |  |  |
| ASI16 | | Post-intervention | 1.24 [2.16, 4.65] | 0.72 (417) | | -0.05 [-0.21, 0.10] | |  |  |
|  | | Follow-up | 2.56 [-3.23, 8.35] | 0.87 (336) | | -0.09 [-0.28, 0.11] | |  |  |

*Note.* *all *p*s > .05; †Degrees of freedom vary between measures because participants were not forced to fill-in the entire set of scale (hence, some were occasionally skipped). All models were adjusted for three covariates (age, gender, and treatment credibility); b – mean change difference in treatment versus control group estimate; d – Cohen’s d for between-groups effects; WSAS – Work and Social Adjustment Scale; BDI-II – Beck Depression Inventory-II; ODSIS – Overall Depression Severity and Impairment Scale; OASIS – Overall Anxiety Severity and Impairment Scale; PSWQ – Penn State Worry Questionnaire; ASI16 – Anxiety Sensitivity Index 16

SM Table 2.

Estimates of mean differences between baseline and post intervention*,* respectively, follow-up (within-group effects), for the secondary outcomes which fall outside of the aim of the present report.

| **Variable** | **Baseline vs. Time** | **Self-enhanced 9UP intervention** | | | **9UP active control** | | |
| --- | --- | --- | --- | --- | --- | --- | --- |
|  |  | ***b* [95% CI]** | ***t** *(df)*†** | *d* [95% CI] | *b* [95% CI] | ***b* [95% CI]** | ***t** *(df)*†** |
| *Secondary outcomes* | | | | |  |  |  |
| WSAS | Post-intervention | -7.50 [-9.19, -5.81] | -8.70 (236) | 0.57 [0.39, 0.74] | -7.25 [-9.35, -5.15] | -6.77 (238) | 0.79 [0.59, 1.00] |
|  | Follow-up | -8.91 [-11.19, -6.64] | -7.68 (236) | 0.45 [0.29, 0.62] | -8.80 [-11.83, -5.77] | -5.69 (237) | 0.70 [0.50, 0.89] |
| BDI-II | Post-intervention | -13.85 [-15.90, -11.80] | -13.23 (251) | 0.95 [0.75, 1.16] | -12.93 [-15.21, -10.65] | -11.11 (246) | 1.15 [0.93, 1.37] |
|  | Follow-up | -15.87 [-18.47, -13.27] | -11.98 (251) | 0.78 [0.59, 0.97] | -14.82 [-17.94, -11.71] | -9.32 (246) | 1.04 [0.83, 1.25] |
| ODSIS | Post-intervention | -3.58 [-4.46, -2.70] | -7.96 (239) | 0.68 [0.47, 0.88] | -3.36 [-4.27, -2.46] | -7.29 (236) | 0.72 [0.52, 0.92] |
|  | Follow-up | -4.07 [-5.25, -2.89] | -6.76 (239) | 0.48 [0.29, 0.67] | -3.56 [-4.89, -2.22] | -5.22 (236) | 0.61 [0.42, 0.80] |
| OASIS | Post-intervention | -3.77 [-4.53, -3.01] | -9.76 (239) | 0.72 [0.52, 0.92] | -3.51 [-4.32, -2.69] | -8.42 (237) | 0.89 [0.68, 1.11] |
|  | Follow-up | -4.30 [-5.31, -3.28] | -8.31 (239) | 0.77 [0.56, 0.97] | -4.88 [-6.06, -3.69] | -8.05 (237) | 0.76 [0.55, 0.96] |
| PSWQ | Post-intervention | -10.37 [-12.20, -8.53] | -11.09 (246) | 0.60 [0.42, 0.78] | -8.08 [-10.34, -5.83] | -7.03 (242) | 0.96 [0.76, 1.17] |
|  | Follow-up | -11.87 [-14.24, -9.50] | -9.80 (246) | 0.52 [0.34, 0.69] | -9.61 [-12.66, -6.56] | -6.18 (242) | 0.85 [0.65, 1.04] |
| ASI16 | Post-intervention | -9.08 [-11.54, -6.61] | -7.22 (237) | 0.60 [0.40, 0.79] | -7.80 [-10.16, -5.44] | -6.48 (236) | 0.66 [0.46, 0.86] |
|  | Follow-up | -13.52 [-16.84, -10.20] | -7.98 (237) | 0.59 [0.39, 0.78] | -11.30 [-14.79, -7.81] | -6.34 (236) | 0.73 [0.53, 0.93] |

*all *p*s > .05; †Degrees of freedom vary between measures because participants were not forced to fill-in the entire set of scale (hence, some were occasionally skipped). All models were adjusted for three covariates (age, gender, and treatment credibility); d – Cohen’s d for within-group effects; WSAS – Work and Social Adjustment Scale; BDI-II – Beck Depression Inventory-II; ODSIS – Overall Depression Severity and Impairment Scale; OASIS – Overall Anxiety Severity and Impairment Scale; PSWQ – Penn State Worry Questionnaire; ASI16 – Anxiety Sensitivity Index 16.

SM Table 3.

Observed means and estimated marginal means for all measurement occasions

| Variable | Group | Time | *n* | *OM* | *SD* | *EMM* | *SE* |
| --- | --- | --- | --- | --- | --- | --- | --- |
| *Primary outcome* | | | | | | | |
| PHQ9 | Intervention | Pre-test | 138 | 13.51 | 6.04 | 13.73 | 0.65 |
|  |  | Post-test | 75 | 7.39 | 5.75 | 7.84 | 0.81 |
|  |  | Follow-up | 33 | 5.69 | 5.96 | 7.39 | 1.06 |
|  | Control | Pre-test | 139 | 14.22 | 6.26 | 14.43 | 0.68 |
|  |  | Post-test | 75 | 6.97 | 4.84 | 8.12 | 0.80 |
|  |  | Follow-up | 37 | 5.57 | 5.04 | 6.69 | 0.97 |
| GAD7 | Intervention | Pre-test | 137 | 11.67 | 5.09 | 11.52 | 0.58 |
|  |  | Post-test | 75 | 6.65 | 5.74 | 6.75 | 0.71 |
|  |  | Follow-up | 33 | 5.12 | 5.17 | 6.27 | 0.92 |
|  | Control | Pre-test | 139 | 12.22 | 5.47 | 12.52 | 0.60 |
|  |  | Post-test | 73 | 5.75 | 4.53 | 6.43 | 0.73 |
|  |  | Follow-up | 36 | 5.50 | 5.37 | 6.29 | 0.90 |
| SPIN | Treatment | Pre-test | 139 | 32.44 | 14.55 | 29.95 | 1.58 |
|  |  | Post-test | 77 | 24.55 | 13.32 | 21.78 | 1.89 |
|  |  | Follow-up | 35 | 22.06 | 15.52 | 19.27 | 2.36 |
|  | Control | Pre-test | 139 | 33.75 | 14.30 | 32.79 | 1.66 |
|  |  | Post-test | 75 | 21.93 | 14.06 | 21.48 | 1.86 |
|  |  | Follow-up | 39 | 20.08 | 13.86 | 17.90 | 2.12 |
| PDSS-SR | Treatment | Pre-test | 138 | 5.91 | 5.93 | 5.61 | 0.61 |
|  |  | Post-test | 75 | 3.81 | 4.78 | 3.37 | 0.71 |
|  |  | Follow-up | 33 | 2.00 | 3.44 | 2.05 | 0.88 |
|  | Control | Pre-test | 138 | 6.99 | 6.24 | 7.52 | 0.66 |
|  |  | Post-test | 75 | 3.16 | 4.34 | 3.99 | 0.75 |
|  |  | Follow-up | 38 | 2.11 | 3.59 | 3.22 | 0.89 |
| *Secondary outcome* | | | | | | | |
| WSAS | Treatment | Pre-test | 141 | 17.65 | 8.23 | 17.03 | 9.78 |
|  |  | Post-test | 74 | 10.51 | 8.47 | 9.78 | 1.19 |
|  |  | Follow-up | 30 | 8.87 | 8.63 | 8.23 | 1.65 |
|  | Control | Pre-test | 140 | 17.26 | 8.75 | 17.50 | 1.01 |
|  |  | Post-test | 70 | 8.97 | 8.09 | 10.00 | 1.18 |
|  |  | Follow-up | 34 | 7.44 | 8.03 | 8.59 | 1.41 |
| BDI-II | Treatment | Pre-test | 140 | 24.88 | 10.17 | 24.71 | 1.12 |
|  |  | Post-test | 78 | 11.06 | 10.42 | 11.78 | 1.38 |
|  |  | Follow-up | 36 | 9.19 | 9.37 | 9.98 | 1.78 |
|  | Control | Pre-test | 139 | 25.68 | 11.31 | 25.15 | 1.24 |
|  |  | Post-test | 77 | 10.42 | 8.95 | 11.30 | 1.41 |
|  |  | Follow-up | 43 | 8.72 | 9.44 | 9.28 | 1.63 |
| ODSIS | Treatment | Pre-test | 141 | 7.35 | 4.38 | 7.51 | 0.50 |
|  |  | Post-test | 74 | 3.68 | 4.53 | 4.15 | 0.60 |
|  |  | Follow-up | 29 | 2.59 | 3.91 | 3.96 | 0.79 |
|  | Control | Pre-test | 141 | 7.33 | 4.93 | 7.58 | 0.55 |
|  |  | Post-test | 71 | 3.18 | 4.07 | 4.00 | 0.63 |
|  |  | Follow-up | 35 | 3.17 | 4.21 | 3.51 | 0.75 |
| OASIS | Treatment | Pre-test | 141 | 8.36 | 3.89 | 8.01 | 0.43 |
|  |  | Post-test | 74 | 4.91 | 3.67 | 4.51 | 0.52 |
|  |  | Follow-up | 30 | 3.13 | 3.40 | 3.14 | 0.69 |
|  | Control | Pre-test | 141 | 8.09 | 4.19 | 8.09 | 0.47 |
|  |  | Post-test | 71 | 4.28 | 3.56 | 4.32 | 0.54 |
|  |  | Follow-up | 35 | 3.63 | 3.79 | 3.79 | 0.64 |
| PSWQ | Treatment | Pre-test | 138 | 61.99 | 9.42 | 59.87 | 1.12 |
|  |  | Post-test | 76 | 54.28 | 10.90 | 51.78 | 1.38 |
|  |  | Follow-up | 36 | 53.06 | 12.74 | 50.26 | 1.75 |
|  | Control | Pre-test | 139 | 62.29 | 9.35 | 61.55 | 1.16 |
|  |  | Post-test | 75 | 51.47 | 10.37 | 51.19 | 1.32 |
|  |  | Follow-up | 40 | 49.53 | 10.55 | 49.68 | 1.53 |
| ASI16 | Treatment | Pre-test | 141 | 30.53 | 13.84 | 28.15 | 1.61 |
|  |  | Post-test | 74 | 23.99 | 15.36 | 20.35 | 1.84 |
|  |  | Follow-up | 29 | 19.83 | 15.03 | 16.86 | 2.27 |
|  | Control | Pre-test | 141 | 30.35 | 13.77 | 30.00 | 1.59 |
|  |  | Post-test | 70 | 19.91 | 12.46 | 20.93 | 1.82 |
|  |  | Follow-up | 34 | 16.18 | 13.44 | 16.48 | 2.15 |
| SCCS | Treatment | Pre-test | 137 | 31.59 | 10.85 | 31.83 | 1.22 |
|  |  | Post-test | 75 | 39.28 | 12.26 | 38.66 | 1.49 |
|  |  | Follow-up | 33 | 44.18 | 11.79 | 41.05 | 1.92 |
|  | Control | Pre-test | 139 | 32.95 | 10.72 | 32.62 | 1.21 |
|  |  | Post-test | 72 | 42.28 | 11.10 | 40.63 | 1.39 |
|  |  | Follow-up | 36 | 44.50 | 8.86 | 43.07 | 1.63 |
| NGSE | Treatment | Pre-test | 137 | 26.19 | 7.57 | 26.53 | 0.85 |
|  |  | Post-test | 75 | 31.36 | 7.82 | 31.40 | 1.01 |
|  |  | Follow-up | 32 | 32.66 | 6.65 | 31.56 | 1.26 |
|  | Control | Pre-test | 139 | 25.71 | 8.35 | 25.62 | 0.82 |
|  |  | Post-test | 72 | 32.61 | 4.78 | 32.13 | 1.00 |
|  |  | Follow-up | 35 | 31.46 | 6.79 | 30.84 | 1.26 |
| USAQ | Treatment | Pre-test | 138 | 67.41 | 16.71 | 69.73 | 2.01 |
|  |  | Post-test | 73 | 82.82 | 21.37 | 84.33 | 2.38 |
|  |  | Follow-up | 29 | 83.14 | 19.92 | 84.30 | 3.06 |
|  | Control | Pre-test | 141 | 67.17 | 17.99 | 66.99 | 2.08 |
|  |  | Post-test | 71 | 83.76 | 18.95 | 80.83 | 2.41 |
|  |  | Follow-up | 34 | 86.85 | 19.18 | 84.29 | 2.89 |
| RSES | Treatment | Pre-test | 141 | 23.41 | 5.31 | 23.68 | 0.61 |
|  |  | Post-test | 74 | 27.77 | 5.57 | 27.70 | 0.73 |
|  |  | Follow-up | 29 | 29.10 | 6.95 | 28.36 | 0.95 |
|  | Control | Pre-test | 141 | 23.41 | 5.56 | 23.36 | 0.65 |
|  |  | Post-test | 71 | 29.13 | 5.58 | 28.44 | 0.76 |
|  |  | Follow-up | 34 | 29.44 | 5.99 | 29.23 | 0.91 |
| SCS | Treatment | Pre-test | 137 | 62.12 | 15.77 | 65.04 | 2.04 |
|  |  | Post-test | 75 | 80.21 | 22.43 | 83.18 | 2.56 |
|  |  | Follow-up | 32 | 43.81 | 11.79 | 85.07 | 3.45 |
|  | Control | Pre-test | 139 | 60.86 | 17.52 | 62.03 | 2.15 |
|  |  | Post-test | 72 | 82.01 | 20.69 | 81.70 | 2.58 |
|  |  | Follow-up | 35 | 44.17 | 8.76 | 82.68 | 3.20 |

*Note.* EMM – estimated marginal means; SE – standard error; OM – Observed Mean; SD – standard deviation.
